# Porous ZnCl_2_-Activated Carbon from Shaddock Peel: Methylene Blue Adsorption Behavior

**DOI:** 10.3390/ma15030895

**Published:** 2022-01-25

**Authors:** Hongxia Zhao, Haihong Zhong, Yu Jiang, Huiyu Li, Pinggui Tang, Dianqing Li, Yongjun Feng

**Affiliations:** 1 State Key Laboratory of Chemical Resource Engineering, College of Chemistry, Beijing University of Chemical Technology, Beijing 100029, China; hxzhaochemistry@163.com (H.Z.); hzhong@mail.buct.edu.cn (H.Z.); huiyuli@buct.edu.cn (H.L.); tangpg@mail.buct.edu.cn (P.T.); lidq@mail.buct.edu.cn (D.L.); 2 Beijing Municipal Construction Group Co., Ltd., A40 Xingshikou Road, Haidian District, Beijing 100195, China; jy558833@126.com

**Keywords:** biomass carbon, adsorption, porous structure, ZnCl_2_-activated carbon

## Abstract

It is of great interest and importance to resource utilization of waste biomass to produce porous carbon for environmental treatments. Pore structure and properties of the obtained carbon mainly relate to carbonization conditions and biomass types. In this work, a series of porous, biomass-activated carbons (AC) were prepared using shaddock peel, with ZnCl_2_ as a pore-forming agent. The effect of carbonization temperature and the mass ratio between ZnCl_2_ and shaddock peel were thoroughly investigated. The material composition, surface chemical properties, and surface structures of samples were carefully characterized. The specific surface area and adsorption capacity to methylene blue (MB) of adsorbents were changed with the carbonization temperature and the mass ratios between ZnCl_2_ and shaddock peel; when the temperature was at 1000 °C and the mass ratio was equal to 2:1, the resulting adsorbent had the largest specific surface area of 2398.74 m^2^/g and average pore size of 3.04 nm, which showed the highest adsorption capacity to MB to be 869.57 mg/g. The adsorption processes of biomass AC adsorbent matched the pseudo-second-order kinetic model and Langmuir isotherm model. This efficient and environmentally friendly biomass AC adsorbent from shaddock peel, activated by ZnCl_2_, is a promising candidate for the treatment of water pollution.

## 1. Introduction

Organic dyes have greatly enriched human life, with widespread applications in textile, papermaking, leather [1], and printing industries [2]. Due to excessive use and uncontrollable discharge, dye pollution is threatening human health and the ecological system. According to statistics, ca. 7 × 10^5^ tons of dye is produced in the world every year [3]. Among them, methylene blue (MB), a heterocyclic aromatic chemical compound (C_16_H_18_C_l_N_3_S_3_H_2_O), is one kind of toxic cationic dye [4] that not only cause diseases, such as heart disease, tissue necrosis, emesis, shock, and others [5], but also has a negative impact on aquatic animals and plant growth due to the reduced photosynthesis [6]. Therefore, it is critical to limit new discharges and remove existing pollution from water bodies using various technologies.

Many technologies, such as electrochemical [7], photoelectrochemical [8], microbial degradation [9], ions exchange [10,11], Fenton reaction [2], membrane separation [12], adsorption [13], and so on, have been developed to remove organic dyes from polluted water. In comparison, adsorption technology is widely used due to its low cost, ease of operation, and high efficiency [14]. Metal oxides [15], carbon materials [16], metalorganic frameworks (MOF) [17], graphene [18], zeolites [19], and polymers [20] have all been used as adsorbents in the treatment of polluted water. Among them, activated carbon (AC) is one of the most commonly used adsorbents, which is one kind of low-cost adsorbent, and the corresponding adsorption performance is primarily determined by the carbon pore structure, which is derived by the carbon resource and carbonization process. Recently, shaddock peel, a type of biomass resource, has piqued the interest of AC investigators due to its abundant supply, low cost, and abundant functional groups [21]. Various types of carbon adsorbents have been synthesized from shaddock peel and used in the energy storage [22,23], gas detection [24], catalysis [25], and adsorption fields [26,27,28]. Usually, the chemical activation method can contribute to the porous structure and chemical properties of carbon surface. KOH [29], H_2_SO_4_ [30], HNO_3_ [31], CaCO_3_ [32,33,34], and so on, have been used as activating agents for the preparation of biomass carbon. Recently, ZnCl_2_ has attracted increasing interest as a high-performance chemical activation agent for fabricating porous carbon materials from various biomass, because ZnCl_2_ acts as a Lewis acid and can be used as a dehydration agent to selectively remove the H and O from biomass to restrain the formation of tar and contribute to the formation of high surface area and porous structure [35,36]. As examples, ZnCl_2_ was used to activate olive solid waste to produce AC with a 10 times higher specific surface area of 1480 m^2^/g and enhanced nitrate adsorption capacity of 5.5 mg/g [37]; ZnCl_2_ was used to activate coconut shell to produce active, magnetic, activated carbon with a more than 275 times improved specific surface area of 935.46 m^2^/g, and an increased maximum adsorption capacity of 156.25 mg/g for MB [38]. Therefore, ZnCl_2_ is a potential activating agent to improve the specific surface area and porosity of carbon materials. It is of great necessity and importance to investigate the influence of ZnCl_2_ in the carbonization of shaddock. Particularly, the adsorption performance of these adsorbents, activated by ZnCl_2_, remains to be improved; moreover, optimizing the pore structure of carbon from shaddock peel and the enhancement of its adsorption performance remains a significant challenge, and few reports on the activation behavior of ZnCl_2_, based on shaddock peel, have been published to date.

In this study, a series of porous ACs were prepared from shaddock peel with ZnCl_2_ as an activating agent. The effects of carbonization and ZnCl_2_ dosage were investigated to get porous structure activated carbon adsorbents. The obtained AC adsorbents with high specific surface area and porous structure were applied for the removal of MB in aqueous solution, and the kinetic and isotherm adsorptions were carefully investigated. The carbonization temperature and ZnCl_2_ dosage are important in optimizing pore structure and enhancing adsorption performance. The use of shaddock peel as a carbon precursor, and of ZnCl_2_ as an activating agent, is a low-cost and environmentally friendly method for practical applications of sewage treatment.

## 2. Materials and Methods

### 2.1. Materials

The zinc chloride (ZnCl_2_) used in this study was of analytical grade, received from Beijing Tongguang Fine Chemical Co., and was used without further purification. Deionized water was used throughout.

### 2.2. Preparation of Activated Carbon

A series of porous AC adsorbents were prepared using shaddock peel as the carbon resource and ZnCl_2_ as the activation agent, with carbonization temperatures ranging from 600 to 1000 °C, and the mass ratios of ZnCl_2_ to shaddock peel at 0 to 6:1. Prior to carbonization, shaddock peel without yellow skin was washed with deionized water, cut into ca. 1 × 1 cm^2^ pieces, and dried in a 90 °C oven at for 12 h. For instance, 10.00 g ZnCl_2_ was dissolved in 150 mL deionized water to form a ZnCl_2_ solution, and then 5.00 g shaddock peel was added to the above solution. The resulting suspension was then kept for another 4 h with vigorous magnetic stirring. Later, the mixed suspension was dried in an oven at 90 °C for another 12 h. The dried sample was placed in a tube furnace under N_2_ atmosphere and carbonized at 800 °C for 2 h with a heating rate of 5 °C/min. The obtained biomass carbon was washed with deionized water, until no Cl^−^ was detected using a 1 wt.% AgNO_3_ aqueous solution. It was ground into a 100 μm powder by a mortar, and collected as 2:1-800, indicating that the collected AC sample was synthesized with a ZnCl_2_ to shaddock mass ratio of 2:1 and a carbonization temperature of 800 °C. Some samples were fabricated at different carbonization temperatures ranging from 600 to 1000 °C at a mass ratio 2:1, following the same procedure, and recoded as 2:1-600, 2:1-700, 2:1-800, 2:1-900, 2:1-1000. Others were performed at 1000 °C with different mass ratios between ZnCl_2_ and shaddock peel, such as 0 (without ZnCl_2_), 1:1, 2:1, 4:1, and 6:1, and labeled as 0-1000, 1:1-1000, 2:1-1000, 4:1-1000, and 6:1-1000, respectively.

### 2.3. Characterization

Crystalline structures were characterized on Bruker D8 Advance powder X-ray diffractometer (Cu Kα1 radiation, λ = 0.15406 nm) from 10 to 70°/2θ at a scan speed of 10° min^−1^. Morphologies were captured using a Hitachi S-4700 scanning electron microscope (SEM) operating at 30 kV, which was also used to record the elemental mapping and spectrum of energy dispersive X-ray spectrometry (EDXS). The transmission electron microscopy (TEM) images were obtained on a HITACHI HT 7700 transmission electron microscope with an accelerating voltage of 100 kV. Specific surface area and pore properties were calculated based on low-temperature N_2_ adsorption–desorption isothermal curves, recorded at 77 K on Micromeritics ASAP 2460 (Norcross, GA, USA), where the specific surface area was evaluated by the Brunauer–Emmett–Teller (BET) method from the adsorption curve, and both the pore size distribution and the pore volume were analyzed using the density functional theory (DFT) method. Micromeritics Auto Pore IV 9500 (Norcross, GA, USA) was used to characterize the macroporous structure. Fourier transform infrared spectra (FT-IR) curves were collected from Bruker Vector 22 spectrophotometer (Karlsruhe, Germany) with mass ratio between sample and potassium bromide at 1:100 following homogeneous mixture.

### 2.4. Batch Adsorption Experiments

The adsorption kinetic experiments on the fabricated AC towards MB were performed in 100 mL conical beakers in a thermostated shaker at 30 °C. Typically, 0.020 g AC powder was dispersed in 40 mL MB aqueous solution with initial concentration of 500 mg/L at nature pH with a shaking speed of 150 rpm, then 1.0 mL of the suspension was extracted after certain time intervals (e.g., 5, 10, 20, 30, 45, 60, 120, 180, 240, 300, 360, and 420 min) through a microfiltration membrane (Φ = 0.22 μm, pore diameter) and the MB concentration in the filtrate was tested by UV-vis spectrophotometer at λ_max_ = 664 nm. The adsorption quantity of the AC towards MB at time t, q_t_ (mg/g) was calculated by the following equation:

(1) $$q_{t}=\frac{\left(C_{0}-C_{t}\right)\times V}{m}$$

where C_0_ (mg/L) represents the initial MB concentration, C_t_ (mg/L) is the MB concentration at time t, V (L) is the volume of MB solution, and m (g) refers to the mass of AC.

In addition, the adsorption isotherm experiments of AC to MB were carried out in conical beakers by dispersing 0.01 g of the AC in each 20 mL MB solution with an initial concentration ranging from 300 to 700 mg/L. After 7 h of shaking at a speed of 150 rpm at 30 °C, the concentration of MB in the solution was measured using a UV-vis spectrophotometer at λ_max_ = 664 nm, the adsorption quantity of the AC to MB at equilibrium, q_e_ (mg/g), was calculated as follows:

(2) $$q_{e}=\frac{\left(C_{0}-C_{e}\right)\times V}{m}$$

where C_0_ (mg/L) represents the initial concentration of MB solution, C_e_ is the equilibrium concentration of MB solution, and V (L) and m (g) refer to the volume of MB solution and the mass of the AC, respectively.

All of the adsorption experiments were repeated three times, and the corresponding average values were used for analysis.

## 3. Results and Discussion

### 3.1. Structure and Morphologies

**Figure 1:**
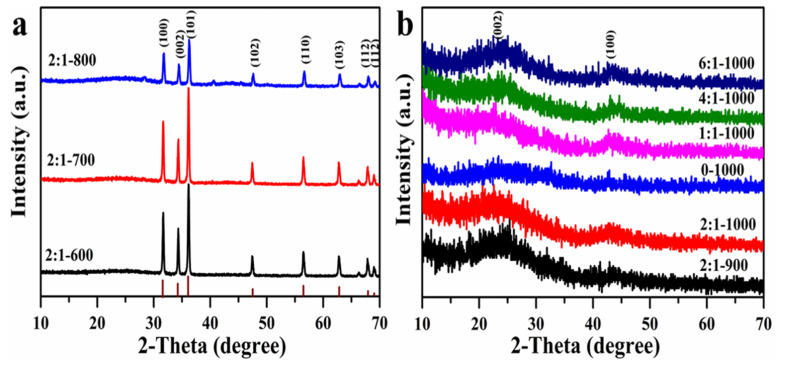
Powder X-ray diffraction patterns (PXRD) of different AC adsorbents prepared at different carbonization temperatures (**a**) and mass ratios (**b**).

A series of porous AC adsorbents were synthesized using shaddock peel as the carbon resource and ZnCl_2_ as the activation agent at various carbonization temperatures (T) ranging from 600 to 1000 °C, and with mass ratios between ZnCl_2_ and shaddock peel scaling from 0 to 6:1. Figure 1 shows powder X-ray diffraction (PXRD) patterns of all the prepared AC adsorbents. As the temperature rises from 600 °C to 800 °C, a series of typical Bragg diffraction peaks for ZnO in the range of 10–70°/2θ were observed, as marked in the graph, which matched well to PDF card No. 79-2205 [39]. Furthermore, the intensity of the related diffraction peak decreased with increasing T from 700 °C to 800 °C, owing to the production of Zn from the reduction between ZnO and carbon [40]. When the T was beyond 900 °C, a broad peak at 2θ = 23° occurred in all AC adsorbents, which was assigned to the (002) plane of carbon carbonized from shaddock peel; a peak at 2θ = 43.8° corresponded to the (100) plane of graphite crystal, and no diffraction peak of ZnO was detected [41], implying complete volatilization of Zn, as reported in the literature [40]. This phenomenon is also verified by the EDXS mapping results in Figure S1 and Table S1; Zn was observed in the samples prepared below 900 °C, when the carbonization increased to 1000 °C, no Zn was observed, and only carbon adsorbents appeared. The 2 diffraction peaks at 23 and 43.8° also existed in all AC samples at different mass ratios when the temperature was fixed at 1000 °C.

**Figure 2:**
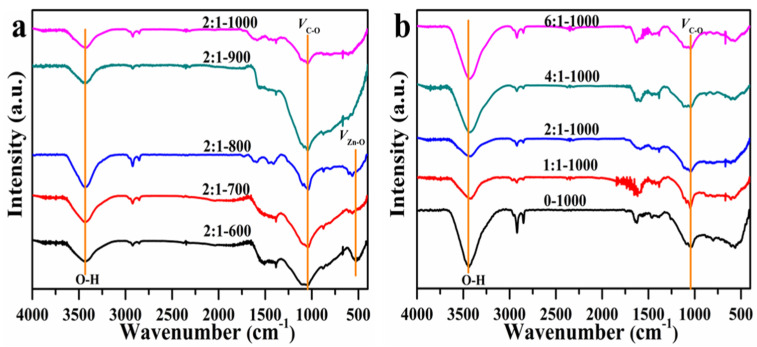
FT-IR curves of different AC adsorbents synthesized with various carbonization temperatures (**a**) and mass ratios (**b**).

Figure 2 furthermore demonstrates the FT-IR spectra of all AC adsorbents in the range of 4000–400 cm^−1^. In all cases, 2 main typical adsorption bonds were observed: 1 at 3433 cm^−1^ attributed to stretching vibration of the hydroxyl group, the other at 1044 cm^−1^, which belonged to the stretching and vibration peak of C-O for alcohols, phenols, or ester groups. For the 3 samples at T = 600–800 °C, 1 adsorption band centered at 529 cm^−1^, which is described as the vibration of the Zn-O bond, which is consistent with the results shown in Figure 1.

**Figure 3:**
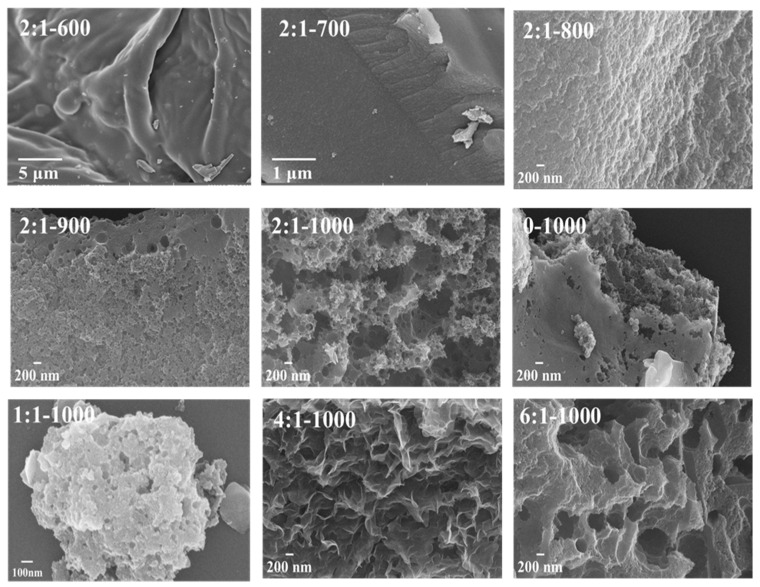
SEM images of AC adsorbents synthesized at various carbonization temperatures (from 600 to 1000 °C) and mass ratios (0, 1:1, 2:1, 4:1, 6:1).

Furthermore, Figure 3 shows SEM images of all the AC samples as a function of carbonization temperature and mass ratios between ZnCl_2_ and shaddock peel, with significantly different morphologies and pore structures. On the one hand, a high carbonization temperature favors the formation of large pore. For example, the carbon surface was smooth and dense at T = 600 and 700 °C; at T from 800 to 1000 °C, the carbon surface varied from crude to porous and the pore size increased with carbonization temperature, with the increase in temperature, the dehydration and gasification of Zn also showed gradually increasing tendency. On the other hand, increasing the use of ZnCl_2_ aided in the production of more macrospores at 1000 °C. For instance, when no ZnCl_2_ was used, there were fewer pores; when the mass ratio was increased from 1:1 to 6:1, more hierarchical pores with varying pore sizes, particularly micrometer-level macrospores, were observed. Besides, Figure S2 shows the TEM images of AC adsorbents. Some differences were observed but it was difficult to distinguish them. We may pay special attention to this issue in the future. Generally, macrospores provide transport channels and increase the exposure of active adsorption sites, since more macrospores may improve adsorption rate and increase adsorption capacity [42]. Enhancing carbonization temperature and a suitable ZnCl_2_ dosage is beneficial for the formation of an optimized porous structure.

### 3.2. Pore Structure

**Figure 4:**
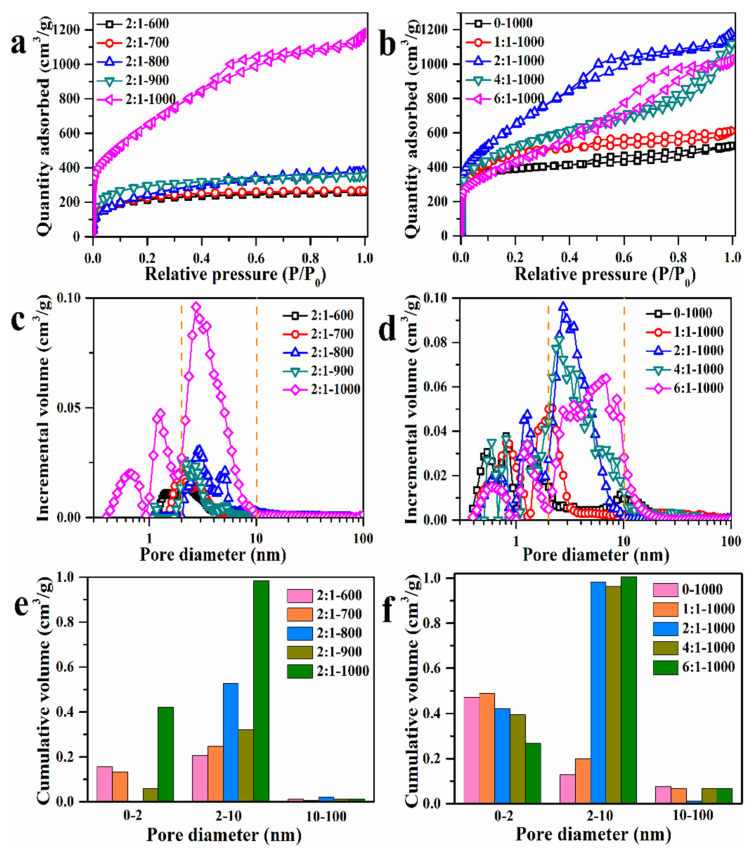
(**a**) N_2_ adsorption–desorption isotherms of various AC adsorbents prepared with different carbonization temperatures and (**b**) mass ratios; (**c**) pore size distribution of various AC adsorbents prepared with different carbonization temperatures and (**d**) mass ratios; (**e**) pore volume distribution of various AC adsorbents prepared with different carbonization temperatures and (**f**) mass ratios.

In the case of porous adsorbents, pore structure is crucial in terms of adsorption rate and maximum adsorption capacity, which are related to pore size and size distribution, as well as surface area. Figure 4 further displays the low-temperature nitrogen adsorption–desorption isotherm curves of AC adsorbents determined at 77 K and a pore diameter distribution graph, as calculated based on the desorption curve from the DFT method. Table 1 also lists the corresponding BET results derived from the desorption curves. According to IUPAC, the isotherm curves of 2:1-600, 2:1-700, 2:1-800, and 2:1-900 (c.f., Figure 4a,b) exhibit a typical IV with a H3 hysteresis loop, indicating that the pore structures were irregular; the isotherm curves in Figure 4c,d show a typical IV with an H4 hysteresis loop for 0-1000, 1:1-1000, 2:1-1000, 4:1-1000, and 6:1-1000, indicating that the pore structures were mainly composed of micropores and mesopores, as observed from the calculated pore diameter distribution [43]. In the case of the 2:1 T samples, the specific surface area increased from 764.30 to 2398.74 m^2^/g and the pore volume increased from 0.40 to 1.82 cm^3^/g as the temperature rose from 600 to 1000 °C; for the mass ratios ranging from 0 to 6:1 at 1000 °C, the specific surface area first increased from 1280.51 m^2^/g to 2398.74 m^2^/g and then decreased to 1560.85 m^2^/g—the optimized mass ratio was 2:1, based on specific surface area and pore volume. In addition, the pore diameter distribution of all the adsorbents can be divided into three ranges: (1) 0.3–2, (2) 2–10, and (3) 10–100 nm, as shown in Figure 4e,f.

The calcined temperature is important in forming richly porous structures before the volatilization temperature of Zn metal, and the addition of ZnCl_2_ favors the formation of mesopores and macropores after the volatilization temperature. For example, the pore diameter of the adsorbents was mainly located at the range of 2–10 nm and expanded to 0.3–2 nm at 1000 °C, which is favorable for the increase in specific surface area and adsorption quantity. The adsorbents synthesized at 1000 °C with a mass ratio of 2:1 exhibited the highest specific surface area of 2398.74 m^2^/g with an average pore diameter of 3.04 nm. To some extent, the high specific surface area and appropriate pore diameter distribution will contribute to the adsorption process.

**Figure 5:**
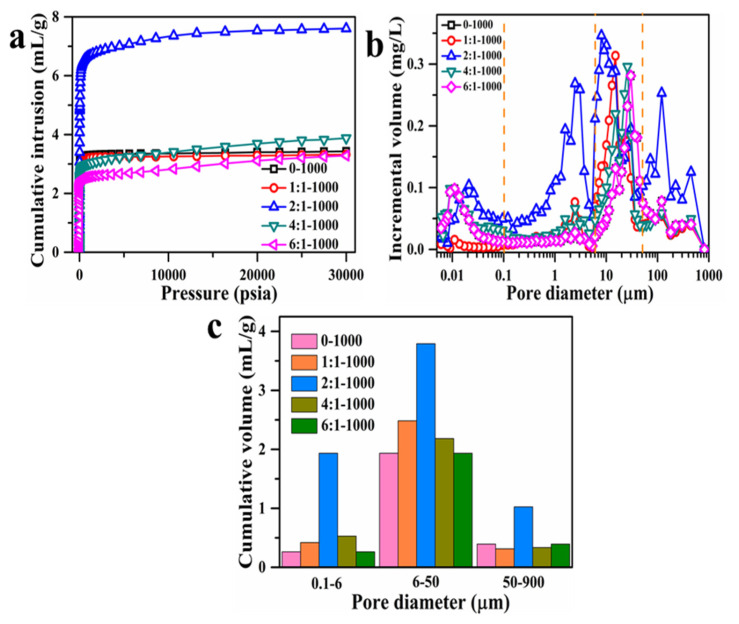
The variation of cumulative intrusion with pressure (**a**), macropore distribution (**b**), and pore volume distribution (**c**) of AC adsorbents from 0-1000 to 6:1-1000 with different mass ratios at 1000 °C.

As shown in Figure 3, macropore structure existed in AC adsorbents synthesized at 1000 °C with different mass ratios. Therefore, the mercury intrusion method was employed to evaluate the macropore structure of AC adsorbents. The corresponding results and pore parameters are shown in Figure 5 and Table 2. From 0.1 µm to 900 µm, the pore size distribution was divided into 3 sections: (1) 0.1–6 µm, (2) 6–50 µm, and (3) 50–900 µm. The 2:1-1000 sample had the highest Hg intrusion volume of 7.61 mL/g, the strongest porosity of 79.26%, and the maximum cumulative volumes at all 3 pore size sections. Therefore, the 2:1-1000 showed perfect hierarchical porous structure and possessed the largest macroporous volume, which contributed to the adsorption performance on MB, because the macropore exposes more adsorption sites and provides transport channels for adsorbate, thereby accelerating the mass transfer into the inner surface of adsorbent [42].

### 3.3. Adsorption Kinetics

**Figure 6:**
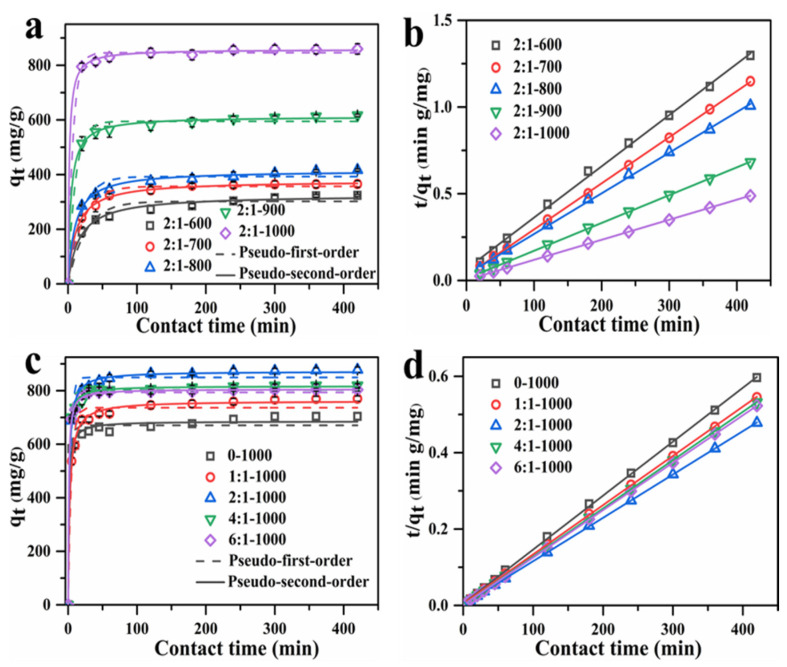
(**a**) The nonlinear fitting of adsorption kinetics and (**b**) linear fitting of pseudo-second-order kinetics of MB for AC adsorbents from different carbonization temperatures. (**c**) The nonlinear fitting of adsorption kinetics and (**d**) linear fitting of pseudo-second-order kinetics of MB for AC adsorbents from different mass ratios.

The adsorption behavior of all AC adsorbents towards MB in aqueous solution, synthesized at different temperatures and mass ratios, was thoroughly investigated. Figure 6a,c show the effects of contact time on the adsorption of all AC adsorbents toward MB. The adsorption quantity increased with contact time, and all samples reached equilibrium when the contact time surpassed 120 min. However, the adsorption rate increased with carbonization temperature, indicating that the carbonization temperature had an effect on the adsorption property.

The nonlinear and linear fitting of kinetics adsorption were described by frequently used, pseudo-first-order and pseudo-second-order adsorption kinetics models, as follows:

(3) $$\mathrm{lg}\left(q_{e}-q_{t}\right)=\mathrm{lg}\left(q_{e}\right)-\frac{k_{1}}{2.303}t$$

(4) $$\frac{t}{q_{t}}=\frac{1}{k_{2}\times q_{e}{}^{2}}+\frac{t}{q_{e}}$$

where the q_t_ (mg/g) represents the adsorption quantity at time t (min), q_e_ (mg/g) is the equilibrium adsorption quantity, and k_1_ (min^−1^) and k_2_ (mg g^−1^ min^−1^) are the adsorption rate constants of pseudo-first-order and pseudo-second-order kinetics models, respectively.

To determine the optimal carbonization temperature, the adsorption kinetic experiments of AC adsorbents toward MB were carried out. Figure 6a,b show the nonlinear fitting results for two kinetic models, as well as the linear fitting results of the pseudo-second-order kinetic model on MB by AC at various carbonization temperatures, while Figure S3a demonstrates the linear fitting of pseudo-first-order kinetic model. The corresponding adsorption kinetic parameters are listed in Table 3 and Table S2. According to the adsorption kinetic parameters, the pseudo-second-order kinetic model is more suitable for the description of the adsorption performance of the AC adsorbents, since it displays a higher R^2^ value than the pseudo-first-order kinetic model, which is better suited to describe the adsorption behavior of adsorbents synthesized at different temperatures. In addition, the theoretical adsorption quantity values (q_e,cal_) of the pseudo-second-order kinetic model are closer to the experimental values (q_e,exp_) than that of the pseudo-first-order model. The adsorption rate increased as the carbonization temperature rose from 600 to 1000 °C, and the 2:1-1000 AC adsorbent reached equilibrium adsorption sooner than that at other temperatures. Moreover, the calculative adsorption quantity increased from 325.87 to 870.37 mg/g as the temperature rose from 600 to 1000 °C, which was in accordance with the increased specific surface area from 764.30 m^2^/g to 2398.74 m^2^/g. More adsorption active sites for MB were provided by the higher specific surface area and more porous structure.

Furthermore, the mass ratio of AC adsorbents at 1000 °C was optimized. Here, Figure 6c,d exhibit the nonlinear and linear fitting results of pseudo-second-order adsorption kinetic curves of AC adsorbents synthesized from different mass ratios, respectively, while Figure S3b displays the linear fitting of pseudo-first-order kinetic model. Table 4 and Table S3 list the calculated adsorption kinetic parameters. The adsorption quantity increased with time and the adsorption reached an equilibrium at ca. 100 min. Based on the R^2^ values of the two models, the pseudo-second-order model matched the adsorption kinetic process more closely, and the theoretical adsorption quantity values (q_e,cal_) of the pseudo-second-order kinetic model were closer to the experimental values (q_e,exp_). The equilibrium adsorption quantity firstly increased with the mass ratios from 0 to 2:1, then decreased from 2:1 to 6:1. Among the 5 samples investigated, the largest equilibrium adsorption capacity of 870.37 mg/g was achieved at a mass ratio of 2:1, which was consistent with the results of specific surface area, so the appropriate mass ratio between ZnCl_2_ and shaddock peel was determined at 2:1.

### 3.4. Adsorption Isotherm

The adsorption isotherm experiments on MB of AC adsorbents synthesized with different mass ratios were carried out, as described in the experimental section. The nonlinear and linear adsorption isotherm fitting results were described by the widely used Langmuir (5) and Freundlich (6) isotherm models.

(5) $$\frac{C_{e}}{q_{e}}=\frac{1}{K_{L}q_{m}}+\frac{C_{e}}{q_{m}}$$

(6) $$\mathrm{lg}q_{e}=\mathrm{lg}K_{F}+\frac{1}{n}\mathrm{lg}C_{e}$$

where q_e_ (mg/g) and q_m_ (mg/g) are the equilibrium and maximum adsorption quantity, C_e_ (mg/L) is the concentration at equilibrium time, K_L_ (L/mg) and K_F_ are the Langmuir and Freundlich adsorption constants, respectively, and n is the adsorption intensity.

**Figure 7:**
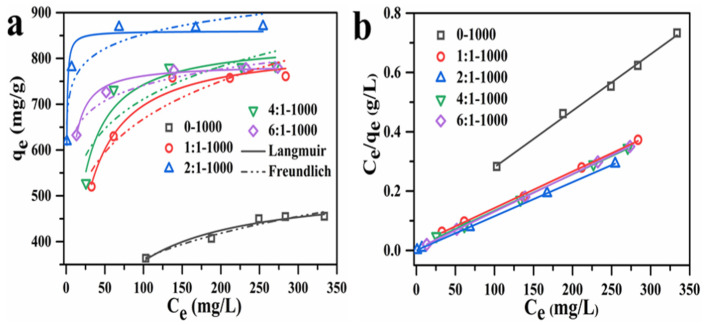
(**a**) The nonlinear fitting of adsorption isotherm; linear fitting of (**b**) Langmuir isotherm for AC adsorbents, prepared with different mass ratios.

Figure 7 shows the nonlinear and linear fitting results of the Langmuir and Freundlich isotherm models and Figure S4 describes the linear fitting of Freundlich isotherm for AC adsorbents with varying mass ratios. Table 5 and Table S4 list the adsorption isotherm parameters of nonlinear and linear adsorption isotherm. The adsorption quantity increased with the increase in equilibrium concentration from 13.31 to 333.79 mg/g, then remained constant. This is due to the fact that the initial concentration was the primary driving force for breaking through the mass transfer resistance between the solid and liquid phases. Because a low concentration of MB cannot occupy all the adsorption sites in the early stage of adsorption, the adsorption quantity was low; as the MB concentration increased, the adsorption quantity increased gradually. However, when the concentration increased to a certain value, the adsorption sites of AC adsorbents became saturated, leading to the unchanged adsorption quantity. According to the adsorption isotherm parameters listed in Table 5 and Table S4, the Langmuir model exhibited higher R^2^ value compared with that of the Freundlich model for both nonlinear and linear fitting modes, implying that the Langmuir model is better suited to describe the adsorption process of the AC adsorbents, demonstrating the monolayer adsorption process of the AC adsorbents [44]. The AC adsorbent synthesized at the mass ratio of 2:1 exhibited a maximum adsorption quantity (q_m_) of 859.81 mg/g. This result was in line with the largest specific surface area and appropriate pore size of the 2:1-1000 AC adsorbent’s honeycomb hierarchical porous morphology, which is beneficial for the adsorption behavior toward MB.

Table 6 lists the specific surface area and adsorption quantity for MB by different adsorbents synthesized from different carbon sources in other publications. As can be seen, the 2:1-1000 AC adsorbent synthesized from shaddock peel in this work shows a superb specific surface area of 2398.74 m^2^/g and a high adsorption capacity of 859.81 mg/g, which is the highest adsorption quantity found among the literature listed. As a result of the high specific surface area, as well as the appropriate pore size, the adsorbent synthesized in our method shows a promising removal performance toward MB.

### 3.5. Post-Analyses Investigation

**Figure 8:**
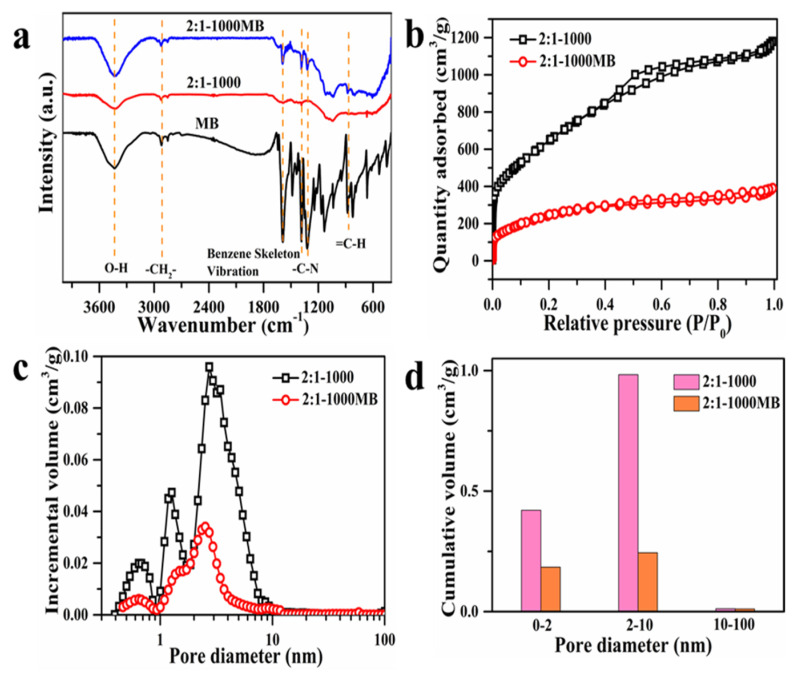
(**a**) FT-IR spectra of MB and 2:1-1000 AC adsorbent before and after the adsorption of MB; (**b**) N_2_ adsorption–desorption isotherm of 2:1-1000 AC adsorbent before and after MB adsorption; (**c**) pore size distribution of 2:1-1000 AC adsorbent before and after the adsorption of MB; (**d**) pore volume distribution of 2:1-1000 AC adsorbent before and after MB adsorption.

In order to further investigate the adsorption behavior toward MB, the 2:1-1000 AC adsorbent was characterized by FT-IR and BET before and after MB adsorption. As depicted in Figure 8a, compared with the FT-IR spectra before adsorption, the FT-IR spectrum of 2:1-1000 adsorbent after adsorption of MB exhibits new characteristic peaks at 873, 1320, and 1381 cm^−1^, which belong to the characteristic adsorption peak of =C-H (aromatics ring) and C-N stretching vibration peaks. Besides, after adsorption, the intensity of the peak at 1595 cm^−1^, attributing to the characteristic adsorption peak of C=C (aromatic ring), increases. These four characteristic peaks also can be found in the same location of the FT-IR spectrum of MB [10]. Therefore, this result indicates that the MB has been successfully adsorbed on the adsorbents via physical adsorption through the abundant porous structure.

Figure 8b–d shows the N_2_ adsorption–desorption isotherm, pore size distribution, and pore volume distribution of 2:1-1000 AC adsorbent. The isotherms of 2:1-1000 before and after MB adsorption show a typical IV with a H4 hysteresis loop, indicating that the micropores and mesopores pores remained in the AC adsorbent, even after MB adsorption. Indeed, after MB adsorption, the specific surface area of 2:1-1000 AC adsorbent decreased from 2398.74 to 899.30 m^2^/g, with a decreased average pore size from 3.04 to 2.67 nm, suggesting that MB had been adsorbed in the inner pore of 2:1-1000 AC adsorbent and occupied the pore space. As shown in Figure 8d, after MB adsorption, the population of the microspore and mesoporous pores decreased dramatically, especially the mesoporous pore. These results reveal that the porous structure, especially the size range of 2–10 nm, makes a significant contribution to the MB adsorption process.

### 3.6. Practical Implications of This Study

Globally, humans have been facing the great challenges of environmental pollution and excessive waste biomass. Novel treatment technologies will be explored including high-efficiency adsorption and separation, which mainly depend on porous adsorbents. It is one of the promising routes of resource utilization of waste biomass to produce porous materials for pollution treatments. Undoubtedly, this work provides a revealing paradigm on the design of efficient adsorbents based on waste biomass in pollution treatment applications.

## 4. Conclusions

In this work, we synthesized a series of activated carbon (AC) adsorbents from shaddock peel by using zinc chloride (ZnCl_2_) as a pore-forming agent, with various carbonization temperatures and mass ratios between ZnCl_2_ and shaddock peel. All of the synthesized AC adsorbents showed good adsorption performance toward MB, and the adsorption process followed the pseudo-second-order kinetics model and Langmuir adsorption isotherm model. The 2:1-1000 AC adsorbent, synthesized at a temperature of 1000 °C and a mass ratio of 2:1, had the highest specific surface area of 2398.74 m^2^/g, a suitable average pore size of 3.06 nm, and the highest MB adsorption capacity of 859.81 mg/g. This demonstrates the importance of a high specific surface area and a proper pore structure for MB adsorption. To summarize, the AC adsorbent, prepared from shaddock peel with ZnCl_2_ as the activator, shows potential for treating water pollution in an economical and efficient manner.

## Figures and Tables

**Table 1:** BET results of various AC adsorbents with different carbonization temperatures and mass ratios.

Samples	Surface Area (m^2^/g)	Average Pore Size (nm)	Pore Volume (cm^3^/g)	Samples	Surface Area (m^2^/g)	Average Pore Size (nm)	Pore Volume (cm^3^/g)
2:1-600	764.30	2.10	0.40	0-1000	1280.51	2.53	0.81
2:1-700	789.10	2.07	0.41	1:1-1000	1663.74	2.27	0.94
2:1-800	881.20	2.65	0.58	2:1-1000	2398.74	3.04	1.82
2:1-900	988.90	2.23	0.55	4:1-1000	1810.08	3.59	1.62
2:1-1000	2398.74	3.04	1.82	6:1-1000	1560.85	4.07	1.31

**Table 2:** The pore parameters of AC adsorbents from different mass ratios at 1000 °C.

Sample	Hg Intrusion Pore Volume (mL/g)	Porosity (%)
0-1000	3.43	74.72
1:1-1000	3.32	67.78
2:1-1000	7.61	79.26
4:1-1000	3.88	75.28
6:1-1000	3.28	74.26

**Table 3:** Nonlinear fitting parameters of pseudo-first-order and pseudo-second-order adsorption kinetics of AC adsorbents with different carbonization temperatures for MB adsorption.

Sample	q_e,exp_ (mg/g)	Pseudo-First-Order		Pseudo-Second-Order			
		q_e,cal_ (mg/g)	k_1_	R^2^	q_e,cal_ (mg/g)	k_2_	R^2^
2:1-600	324	313.55	0.03838	0.7184	325.87	3.436 × 10^−4^	0.8935
2:1-700	366	341.80	0.08742	0.6893	364.01	3.998 × 10^−4^	0.9066
2:1-800	417	373.27	0.01104	0.7854	404.09	4.048 × 10^−4^	0.9469
2:1-900	617	609.43	0.1979	0.6909	615.85	6.104 × 10^−4^	0.9152
2:1-1000	879	849.55	0.2952	0.9809	871.87	7.567 × 10^−4^	0.9979

**Table 4:** Nonlinear fitting parameters of pseudo-first-order and pseudo-second-order adsorption kinetics of biomass carbon adsorbents with different mass ratios for MB adsorption.

Sample	q_e,exp_ (mg/g)	Pseudo-First-Order		Pseudo-Second-Order			
		q_e,cal_ (mg/g)	k_1_	R^2^	q_e,cal_ (mg/g)	k_2_	R^2^
0-1000	704	670.45	0.3701	0.9747	685.56	1.310 × 10^−3^	0.9908
1:1-1000	771	736.28	0.2198	0.9714	762.22	5.541 × 10^−4^	0.9958
2:1-1000	879	849.55	0.2952	0.9809	871.87	7.567 × 10^−4^	0.9979
4:1-1000	822	802.70	0.3849	0.9895	817.13	1.320 × 10^−3^	0.9981
6:1-1000	803	793.32	0.3879	0.9946	805.43	1.490 × 10^−3^	0.9992

**Table 5:** Nonlinear fitting parameters of Langmuir and Freundlich adsorption isotherm of AC adsorbents with different mass ratios for MB adsorption.

Samples	Langmuir Isotherm			Freundlich Isotherm		
	q_m_ (mg/g)	K_L_ (L/mg)	R^2^	K_F_ (mg/g)	n	R^2^
0-1000	523.96	0.02141	0.9420	139.99	0.2067	0.9383
1:1-1000	829.90	0.05261	0.9663	309.22	0.1673	0.8448
2:1-1000	859.81	2.9518	0.9429	663.85	0.05437	0.8535
4:1-1000	841.48	0.07507	0.9167	377.04	0.1378	0.6932
6:1-1000	787.23	0.2988	0.9825	543.48	0.06735	0.9181

**Table 6:** Comparison the adsorption capacity of different adsorbents for MB.

Adsorbent	Activating Agent	Carbonizatin Temperature (°C)	Specific Surface Area (m^2^/g)	Adsorption Capacity (mg/g)	Ref.
Corncob AC	KOH	700	1405.00	636.94	[45]
Coconut AC	NaOH	600	876.14	200.01	[46]
Wood AC	H_3_PO_4_	500	1161.29	159.89	[47]
Soybean dregs AC	ZnCl_2_	500	643.58	225.10	[48]
Walnut shells AC	ZnCl_2_	450	1800.00	315.00	[49]
Banana peel AC	NaOH	400	432.00	232.50	[50]
Palm shell AC	-	-	731.50	163.30	[51]
Peanut shell AC	NaOH	800	868.75	555.60	[52]
Sewage sludge and Coconut shell AC	KOH	700	873.54	623.37	[53]
Magnetic coal-based AC	KOH	1000	1188.00	238.56	[54]
Shaddock peel AC	ZnCl_2_	1000	2398.74	859.81	This work

## Data Availability

Not applicable.

## Supplementary Materials

The following supporting information can be downloaded at: https://www.mdpi.com/article/10.3390/ma15030895/s1, Figure S1: The EDXS mappings of 2:1-600, 2:1-700, 2:1-800, 2:1-900 and 2:1-1000, Figure S2: The TEM images of AC adsorbents synthesized at various carbonization temperatures (from 600 to 1000 °C) and mass ratios (0, 1:1, 2:1, 4:1, 6:1), Figure S3: The linear fitting of pseudo-first-order kinetics for MB by AC adsorbents from (a) different carbonization temperatures and (b) different mass ratio, Table S1: The element percentage of C, O, Zn in adsorbents prepared with different carbonization temperatures, Table S2: Linear fitting parameters of pseudo-first-order and pseudo-second-order adsorption kinetics of biomass carbon adsorbents with different carbonization temperatures for MB adsorption, Figure S4: The linear fitting adsorption isotherm of Freundlich adsorption isotherm for AC adsorbents prepared with different mass ratios, Table S3: Linear fitting parameters of pseudo-first-order and pseudo-second-order adsorption kinetics of AC adsorbents with different mass ratios for MB adsorption, Table S4: Linear fitting parameters of Langmuir and Freundlich adsorption isotherm of AC adsorbents with different mass ratios for MB adsorption.
