# Supplementary material for: Porous ZnCl2-Activated Carbon from Shaddock Peel: Methylene Blue Adsorption Behavior
Source: Materials (Basel). 2022 Jan 25;15(3):895. doi: 10.3390/ma15030895 (PMC8839101; doi:10.3390/ma15030895)
Supplement: Supplementary file 1 [file materials-15-00895-s001.zip › materials-1523175-supplementary.pdf]

materials

Supplementary Information

Porous $ZnCl_2$-Activated Carbon from Shaddock Peel: Methylene Blue Adsorption Behavior

Hongxia Zhao [1], Haihong Zhong [1], Yu Jiang [2], Huiyu Li [1], Pinggui Tang [1], Dianqing Li [1], and Yongjun Feng [1,*]

[1] State Key Laboratory of Chemical Resource Engineering, College of Chemistry, Beijing University of Chemical Technology, Beijing 100029, China.
[2] Beijing Municipal Construction Group Co. Ltd, A40 Xingshikou Road, Haidian District, Beijing, China, 100195.
* Correspondence: yjfeng@mail.buct.edu.cn Fax: +86 10 6442 5385; Phone: +86 10 6443 6992

Citation: Zhao, H.; Zhong, H.; Jiang, Y.; Li, H.; Tang, P.; Li, D.; Feng, Y. Porous $ZnCl_2$-Activated Carbon from Shaddock Peel: Methylene Blue Adsorption Behavior. *Materials* **2022**, *15*, 895. https://doi.org/10.3390/ma15030895

Academic Editor: Avelino Núñez-Delgado

Received: 8 December 2021
Accepted: 18 January 2022
Published: 25 January 2022

Publisher's Note: MDPI stays neutral with regard to jurisdictional claims in published maps and institutional affiliations.

Copyright: © 2022 by the authors. Licensee MDPI, Basel, Switzerland. This article is an open access article distributed under the terms and conditions of the Creative Commons Attribution (CC BY) license (https://creativecommons.org/licenses/by/4.0/)

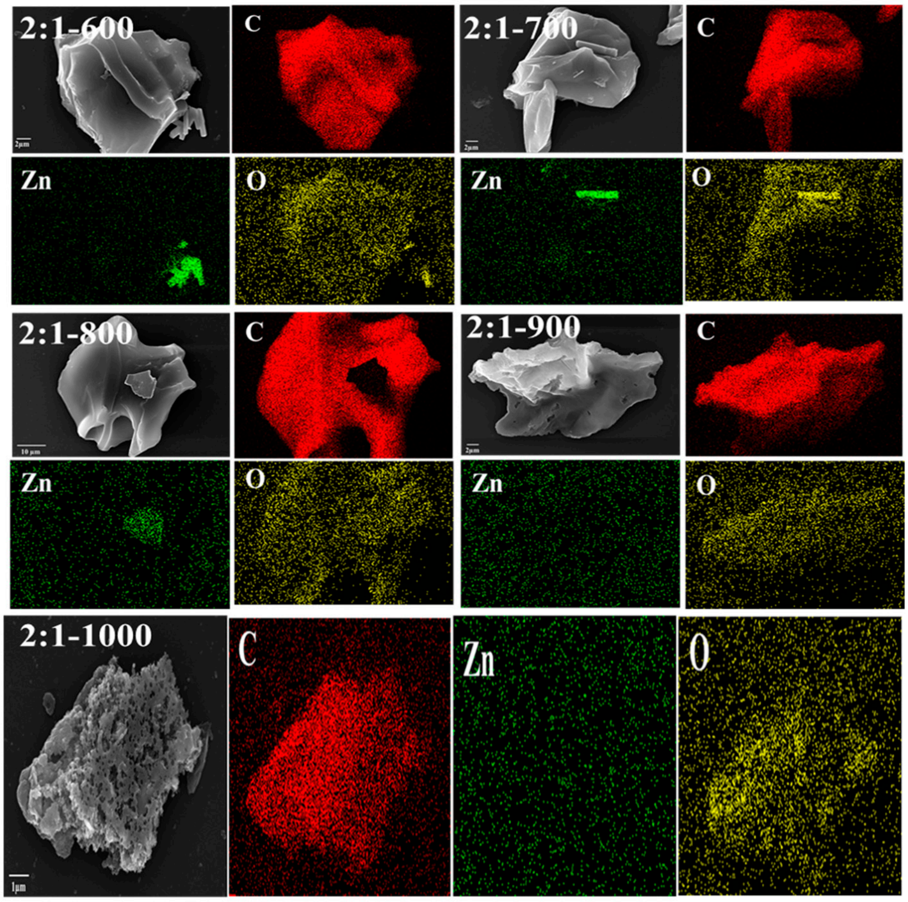

Figure S1. The EDXS mappings of 2:1-600, 2:1-700, 2:1-800, 2:1-900 and 2:1-1000.

Table S1. The element percentage of C, O, Zn in adsorbents prepared wit different carbonization temperatures.

Samples	Element Percentage		
	C (%)	O (%)	Zn (%)
2:1-600	87.94	11.63	0.43
2:1-700	89.15	10.59	0.26
2:1-800	92.62	7.36	0.02
2:1-900	89.78	10.21	0.01
2:1-1000	72.74	27.26	0

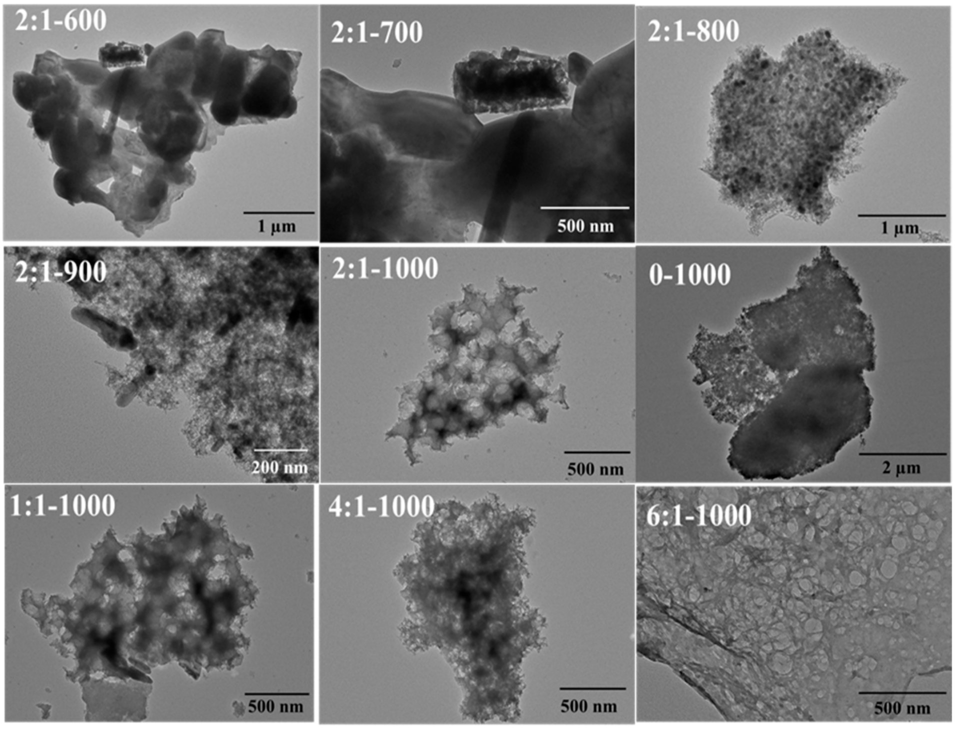

Figure S2. The TEM images of AC adsorbents synthesized at various carbonization temperatures (from 600 to 1000 °C) and mass ratios (0, 1:1, 2:1, 4:1, 6:1).

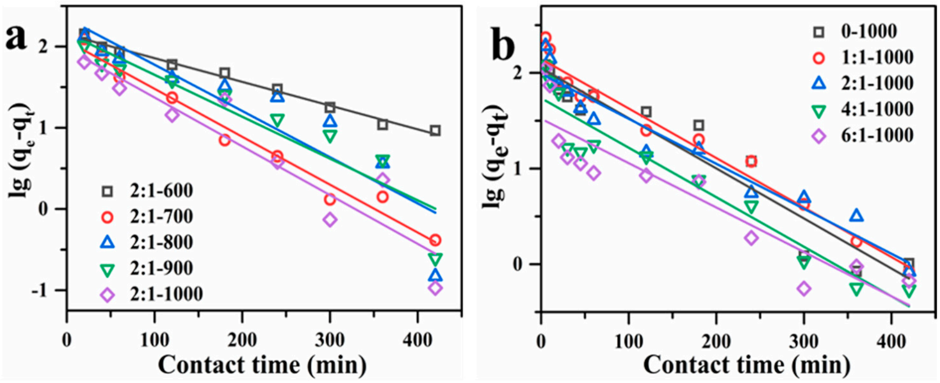

Figure S3. The linear fitting of pseudo-first-order kinetics for MB by the AC adsorbents from (**a**) different carbonization temperatures and (**b**) mass ratios.

Table S2. Linear fitting parameters of the pseudo-first-order and pseudo-second-order adsorption kinetics of biomass carbon adsorbents with different carbonization temperatures for MB adsorption.

Samples	$q_{e, exp}$ (mg/g)	Pseudo-First-Order			Pseudo-Second-Order		
		$q_{e, cal}$ (mg/g)	k_1	R^2	$q_{e, cal}$ (mg/g)	k_2	R^2
2:1-600	324	140.99	0.006725	0.9868	337.84	0.000131	0.9981
2:1-700	366	117.44	0.01359	0.9747	375.94	0.00025	0.9999
2:1-800	417	221.60	0.01310	0.8046	427.35	0.000163	0.9992
2:1-900	617	147.02	0.01186	0.8552	625.00	0.000242	0.9998
2:1-1000	879	94.09	0.01384	0.8667	862.07	0.000424	0.9999

Table S3. Linear fitting parameters of the pseudo-first-order and pseudo-second-order adsorption kinetics of the AC adsorbents with different mass ratios for MB adsorption.

Samples	$q_{e, exp}$ (mg/g)	Pseudo-First-Order			Pseudo-Second-Order		
		$q_{e, cal}$ (mg/g)	k_1	R^2	$Q_{e, cal}$ (mg/g)	k_2	R^2
0-1000	704	111.94	0.01207	0.9157	709.22	0.000385	0.9997
1:1-1000	771	139.86	0.01193	0.9662	775.19	0.000346	0.9999
2:1-1000	879	94.09	0.01384	0.8667	862.07	0.000424	0.9999
4:1-1000	822	54.45	0.01193	0.8787	819.67	0.00093	0.9999
6:1-1000	803	33.18	0.01069	0.8261	806.45	0.001437	0.9999

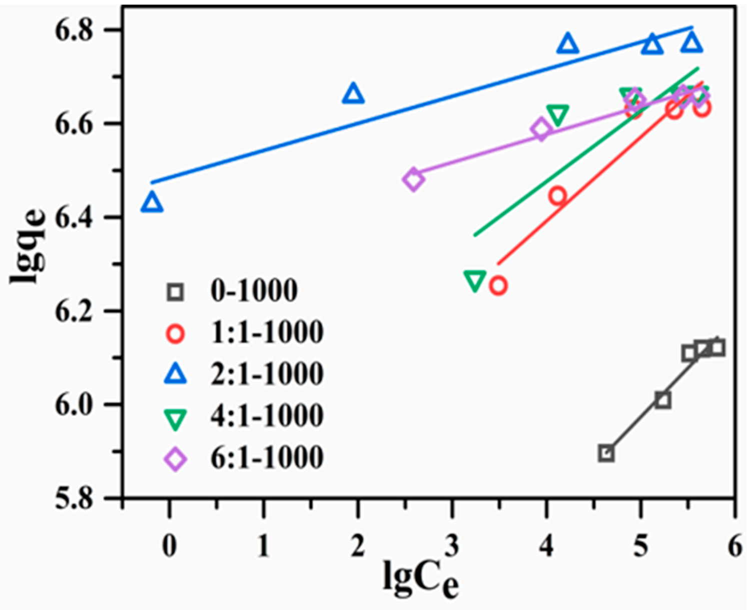

Figure S4. The linear fitting adsorption isotherm of Freundlich adsorption isotherm for the AC adsorbents prepared with different mass ratios.

Table S4. Linear fitting parameters of Langmuir and Freundlich adsorption isotherms of the AC adsorbents with different mass ratios for MB adsorption.

Samples	Langmuir Isotherm			Freundlich Isotherm		
	q_m (mg/g)	K_L (L/mg)	R^2	K_F (mg/g)	n	R^2
0-1000	523.56	0.02122	0.9902	85137.32	4.7897	0.9477
1:1-1000	813	0.06241	0.9902	474307.5	5.5831	0.8662
2:1-1000	869.57	1.8880	0.9999	3054007	17.2951	0.8611
4:1-1000	813.01	0.1132	0.9946	748876.2	6.6428	0.6465
6:1-1000	787.40	0.2906	0.9995	2176256	16.7112	0.9479
